# A step towards sustainable glass fiber reinforced concrete utilizing silica fume and waste coconut shell aggregate

**DOI:** 10.1038/s41598-021-92228-6

**Published:** 2021-06-17

**Authors:** Osama Zaid, Jawad Ahmad, Muhammad Shahid Siddique, Fahid Aslam, Hisham Alabduljabbar, Khaled Mohamed Khedher

**Affiliations:** 1 Department of Civil, Engineering, Military College of Engineering, 24080, Risalpur, National University of Sciences and Technology, Islamabad, Pakistan; 2 Department of Civil Engineering, College of Engineering in Al-Kharj, Prince Sattam Bin Abdulaziz University, Al-Kharj, 11942 Saudi Arabia; 3 Department of Civil Engineering, College of Engineering, King Khalid University, Abha, 62529 Saudi Arabia

**Keywords:** Engineering, Structural materials

## Abstract

Today, it’s getting harder to find natural resources for concrete production. Utilization of the waste materials not just helps in getting them used in concrete, cement, and other construction materials, but also has various secondary advantages, for example, saving in energy, decrease in landfill cost, and protecting climate from pollution. Considering this in the development of modern structural design, utilizing waste materials instead of natural aggregate is a good option to make concrete that is sustainable and eco-friendly. The present research aims to find the impact of adding glass fiber into sustainable concrete made with silica fume, as a partial replacement of cement, and coconut shell added with different ratios as a replacement of coarse aggregate, on concrete mechanical and durability aspects. Various blends were made, with coconut shell as a substitution of coarse aggregates with different ratios. Portland cement was substituted with silica fume at 5%, 10%, 15%, and 20% by cement weight in all concrete blends. The volume ratios of glass fibers utilized in this study were 0.5%, 1.0%, 1.5% and 2.0%. Adding glass fibers increases concrete density to some extent and then marginally reduces the density of coconut shell concrete. When the percentage of glass fibers increases, the compressive, flexural and split tensile strength of coconut shell concrete also increases. From the lab results and SEM images of the present research display that glass fibers might be utilized in coconut shell concrete to enhance its mechanical and durability attributes, to accomplish sustainable concrete with acceptable strength with ease.

## Introduction

Sustainability might be defined as the capacity to retain depleting nonrenewable resources and renewable resources use indefinitely. Sustainability is one of the highest studied yet least comprehended words on the planet. For most of the countries, associations, and individuals who think about its significance, sustainability implies the Earth preservation and critical matters connected to development, for example, stable economic growth, productive usage of resources, poverty elimination, and consistent social advance. Sustainable construction expects to meet present prerequisites for a working situation, housing, and infrastructure without trading off people's capacity in the coming years to address their issues^1^. Researchers in writing^2^ announced that the present phase of the construction industry is not sustainable. Sustainable construction could be accomplished in the industry by substituting normal crushed stone in concrete with strong recycled products. Crushed stone aggregates and fine are around 65–85% of the volume of concrete^3–5^. However, the utilization of conventional coarse aggregates subverts sustainability, as it prompts additional environmental issues^6^. As per the India Farmer's Welfare and Horticulture Ministry, in 2016–2017 over and above 2390 Billion coconuts have been formed in India^7^ this will increment the farming waste accumulation. Thus, the successful usage of agricultural wastes, as a substitute for normal aggregates, contributes to the preservation of depleting resources which is non-renewable, decreases utilization of energy, and also decreases the expenses of construction materials. Authors has utilized a few scrap matters in concrete, with recycled aggregate, fly ash, silica fume waste elastic tire, and GGBFS and waste and glass plastics^8,9^. Moreover, tobacco wastes, coconut shell (CS), rice husk ash, palm shell, pistachio shell, oil palm shell (OPS), utilized to deliver lightweight aggregate concrete. Therefore, the protection of the environment could be accomplished by the best utilization of this type of agricultural wastes^2^.

Lightweight aggregate concrete shows particular points of interest on different kinds of concrete, for example, lighter formwork, less seismic forces, lower dead weight, increased resistance to fire, smaller size foundation, better sound insulation, thermal insulation, and increased resistance to fire. Lightweight concrete for a structure might be delivered by substituting normal crushed stone with specific aggregates which are lightweight, for example, GGBFS, peach shell, pumice extended earth, clinker, and OPS^10^. Similarly, the replacing of normal coarse aggregates with coconut shells has paved the way for structural lightweight concrete^11,12^.The durability aspects of Coconut Shell concrete, for example, absorption, rapid chloride penetrability, color changes, the volume of porous voids, residual strength, and resistance at raised temperatures, are comparable to lightweight concretes^13,14^. Gunasekaran et al.^15^ demonstrated that when subjected to torsion coconut shell concrete beams behaved similarly to normal concrete. The fracture toughness and mechanical attributes of coconut Shell concrete are practically identical to lightweight concrete^8^.

Adding fibrous material enhances concrete structural integrity. A recent study displays that the stresses like tensile and shear that emerge at a various cross-section of lightweight concrete might be reduced by strengthening concrete with glass fiber, natural fibers, and nylon^16–19^. Glass fibers have more resistance to temperature, corrosion resistance, non-flammability, and good strength in tension, but also great sound, heat, and electric padding. Glass fibers could be used in concrete for different purposes, for example, forestalling the coalescence of cracks, crack control, and changing the material behavior by cracks bridging^20,21^. Adding glass fibers to RAC was seen to enhance the tensile strength by 12–19%, and the energy absorption capacity and deformation capacity are also enhanced^22^. Silica fume is a byproduct of manufacturing ferrosilicon or silicon metal composites. Perhaps the most significant use for silica fume is in concrete. Due to its compound and actual attributes, it is an exceptionally passive pozzolana. Concrete that contains silica fume may be durable and could have good strength. Silica fume is acquirable from concrete admixtures providers and, when needed, it is just added into concrete. Silica fume concrete needs special consideration concerning the concrete contractual work^23^. According to a survey conducted in 2019 by Pakistan agricultural research council, the cultivation of coconut in Pakistan is 9917 tons annually, which is a huge number. When people will consume this much coconut it is obvious that coconut shell will cause land pollution when it is discarded in landfills. Now utilizing this coconut shell in concrete as a coarse aggregate is a perfect solution to reduce land pollution and also makes concrete eco-friendly.

In the present study, various mixes were prepared in which natural coarse aggregates were replaced with coconut shell (CS) aggregates in different ratios along with silica fume and glass fibers. Crushed stone coarse aggregate was replaced with coconut shell (CS) aggregate at 15%, 30%, 45%, and 60%. Cement was partially substituted with silica fume at 5%, 10%, 15%, and 20% rates. Glass fiber was utilized at 0. 5%, 1.0%, 1.5% and 2.0% of cement weight. The goal of the present research is to explore the method of making concrete that is both an economically viable option with acceptable strength and environmentally friendly. Mechanical performance like compressive and splitting tensile strength and durability performance like density, water absorption, acid resistance tests, UPV tests, and scanning electron microscope (SEM) tests were performed, to assess concrete sustainable performance.

## Materials

### Cement

In the present research, OPC cement was used, which is type I (42.5) general-purpose cement as per ASTM C 150^24^. The chemical and physical properties of ordinary Portland cement are displayed in Table 1.

**Table 1:** Physical and chemical properties of OPC cement.

Chemical properties	Percentage (%)	Physical properties	Results
CaO	64.7	Size	≤ 75µ
SiO_2_	23.9	Fineness	92%
Al_2_O_3_	8.4	Normal consistency	28%
Fe_2_O_3_	1.7	Initial setting time	38 min
MgO	2.5	Final setting time	412 min
SO_3_	1.2	Specific surface	320 m^2^/kg
K_2_O	2.4	Soundness	1.70%
Na_2_O	0.2	28-days compressive strength	42 MPa

### Glass fibers

Glass fibers of 18 mm were utilized for the current study for all Mixes. Its physical properties are given in Table 2.

**Table 2:** Physical properties of glass fibers.

Physical properties	Results
Length	18 mm
Diameter	0.7 mm
Aspect ratio (L/d)	25.71
Tensile strength	2500 MPa
Density	2.5 g/cm^3^
Young’s modulus	70 GPa
Elongation at break	2.5%

### Aggregates

**Figure 1:**
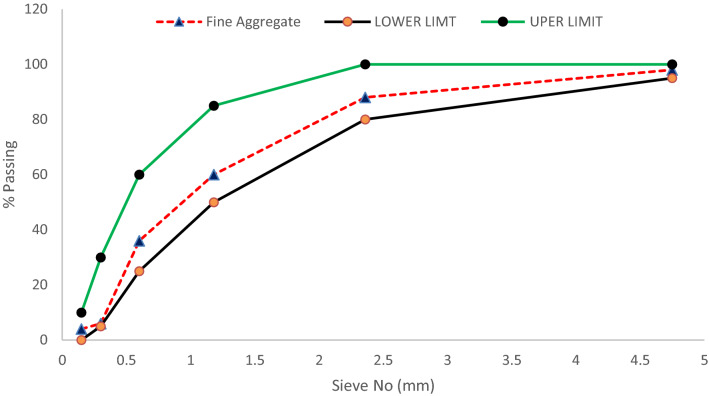
Grading size curve of fine aggregate.

Natural river sand which was passed through sieve no 4.75 mm was used for the study. The sand was washed with water before it was used in concrete to make sure it had no silt content. Sand density was 1586 kg/m^3^ and it was utilized in all the blends. Its properties are provided in Table 3. The particle size distribution of fine aggregate can be seen in Fig. 1.

**Table 3:** Physical properties of coarse, fine and coconut shell aggregate.

Physical property	Coarse aggregate	Fine aggregate	Coconut shell
Bulk density(kg/m^3^)	1720	1586	654
Moisture content	1.25%	1.8%	Nil
Absorption capacity	2.13%	5.28%	0.6
Fineness modulus	4.7	2.93	5.2
Particle size	12.5 mm to 4.75 mm	4.75 mm to 0.75	12.5 mm to 4.75 mm

**Figure 2:**
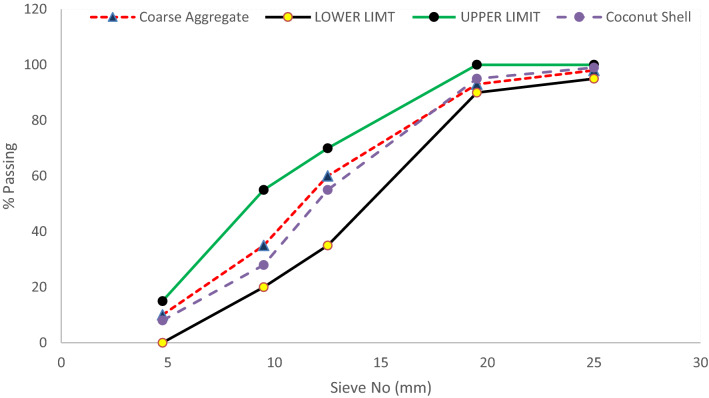
Grading size curve of coarse aggregates.

Coconut shell aggregate had a unit weight of 654 kg/m^3^ and was utilized as a coarse aggregate. The bulk density of crushed stone coarse aggregate was 1720 kg/m^3^, which was used with the adding of Coconut nutshell aggregate in the second mix. The difference between the density of crushed stone coarse aggregate and Coconut nutshell aggregate was 1066 kg/m^3^, which considerably decreases the structural member's self-weight. Physical properties of coconut shell aggregate and conventional coarse aggregate are displayed in Table 3. The coconut shell was acquired from a local coconut seller. The coconut shell was washed; air-dried and was then crushed in Los Angeles abrasion apparatus it was then passed through different sieves size to obtain a size of 4.75 mm to 12.5 mm. Since Coconut shell aggregate absorbs water significantly it was oven-dried to use coconut shell aggregate in SSD (saturated surface dry) conditions. Gradation curve of coarse aggregates (crushed stone aggregate + coconut shell aggregate) is provided in Fig. 2.

### Silica fume

Silica fume was procured from Pak re-rolling mills. Its physical properties can be seen in Table 4.

**Table 4:** Chemical and physical properties of silica fume.

Chemical property	Percentage (%)	Physical property	Results
SiO_2_	87.6	Particle size (average)	≤ 0.2 µm
CaO	2.9	Specific gravity	2.3
Al_2_O_3_	5.7	Color	Light grey
Fe_2_O_3_	1.7	Bulk density (kg/m^3^)	570
MgO	2.5	Solubility	Insoluble
SO_3_	1.9	Specific surface area	22 m^2^/g
K_2_O	2.4		
Na_2_O	0.2		

### Superplasticizer

In the present research, Conplast SP 430 superplasticizer was used. It is included commonly in the scope of 0.6% to 1.3% by cement weight. The utilization of a superplasticizer makes pumpable concrete and concrete which is more workable. In the current investigation, a 0.8% superplasticizer was utilized to control test samples. Conplast SP430 meets with BS 5075, BS: EN 934-2^25^, and with ASTM C494 as Type A and Type F^26^, liable upon measurements utilized. In Table 5 its physical properties are provided.

**Table 5:** Physical properties of superplasticizer.

Property	Result
Color	Brown liquid
Air entrainment	1.48 at 25 °C
Chloride content	Nil to BS 5075/BS: EN934
Specific gravity	1.18

## Methods and mix

For the control sample, we went for M20 grade concrete in which there were no CS aggregates, glass fibers, and silica fume. The complete mix design can be seen in Table 6. Crushed stone coarse aggregate was replaced with coconut Shell (CS) aggregate at 15%, 30%, 45%, and 60%, and glass fibers were utilized at 0. 5%, 1.0%, 1.5% and 2.0% by weight of cement and silica fume was added instead of cement by 5%, 10%, 15% and 20%. For destructive testing, compressive, and split tensile strength at 7 and 28 days were determined and for non-destructive testing, density, water absorption, acid resistance test, and ultrasonic pulse velocity test of concrete were considered at 28 days. The water to cement ratio was kept the same for the study and a mid-range superplasticizer was also added by 0.8% weight of cement to achieve a concrete having an acceptable slump value.

**Table 6:** Mix proportion of concrete (kg/m^3^).

Mix ID	Mix proportion			Cement (kg/m^3^)	Silica fume (SF) (kg/m^3^)	Coarse aggregate (kg/m^3^)	Coconut shell aggregate (CS) (kg/m^3^)	Glass fibers (kg/m^3^)	Sand (kg/m^3^)	Water (kg/m^3^)	Admixture (kg/m^3^)	Slump (mm)
	Cement (C)	Aggregate	Glass fibers (%)									
Control	100% C	100% CA	0	320	0	895	0	0	460	175	2.4	55
Mix 1	C:SF (95:5)	CA:CS (85 :15)	0.50	304	16	760.75	134.25	15.2	460	175	2.4	45
Mix 2	C:SF (90:10)	CA:CS (70 :30)	1.00	288	32	625.5	268.5	28	460	175	2.4	40
Mix 3	C:SF (85:15)	CA:CS (55 :45)	1.50	272	48	492.25	402.75	40.8	460	175	2.4	37
Mix 4	C:SF (80:20)	CA:CS (40 :60)	2.00	256	64	358	537	51.2	460	175	2.4	35

## Interpretation of results

### Destructive tests

#### Compressive strength

Compressive strength is the measure of the greatest compressive loading concrete can withstand. The compressive strength test is completed under the standard procedure of ASTM as ASTM C39/C39M^27^ for cylindrical specimens having standard dimensions as 150 mm diameter and 300 mm length.

**Figure 3:**
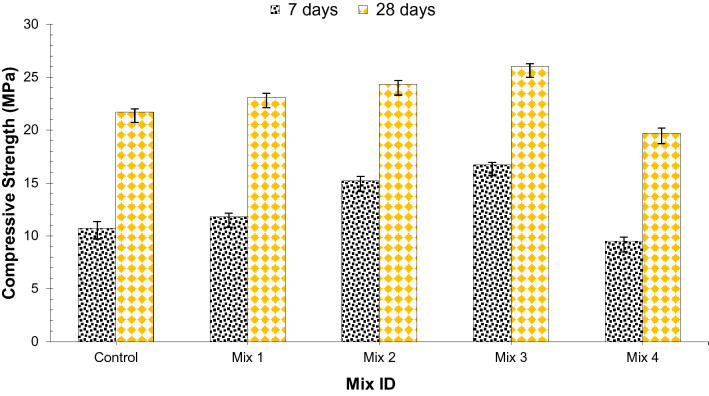
Compressive strength of concrete at 7 and 28 days of curing.

Figure 3 displays the outcomes of the compressive strength test on concrete cylinders with different dosages. The compressive strength of concrete having coconut shell aggregate, silica fume, and glass fibers is increased up to a certain level but then decreased as shown in Fig. 3. Standard deviation and coefficient of variation for compressive strength at 7 and 28 days is presented in Table 7. From the outcome, it can be understood that concrete with 45% CS aggregate, 1.5% glass fibers, and 15% silica fume, showed a much better result than all specimens in terms of compressive strength. Although it is revealed that excess content of coconut shells reduce strength due to the poor bond between cement and coconut shell^8^. The positive response of compressive strength is because of the pozzolanic reaction of SiO_2_ in silica fume with CH of cement producing additional cementitious compounds. It has been also reported that compressive strength considerably improved with silica fume^28^. The extra binder formed by the silica fume reaction with existing lime Ca (OH_2_) permits silica fume concrete to continue to increase strength over time. However, at a higher dosage of silica fume (beyond 15% by weight of cement) strength reduce due dilution effect which leads to alkali-silica reaction due to a higher quantity of unreactive silica available is because of the silica fume high quantity. Furthermore, the positive effect on compressive strength is due to the confinement of the fiber reinforcement on the concrete sample. Compression produces an expansion laterally, and with it, tension and shear. The tension and shear are resisted by the fibers. Therefore, compression is increased. When the percentage of fibers is more this confinement can decrease concrete sample transversal deformation and enhance its compressive strength.

**Table 7:** Standard deviation and coeffiecient of variation of compressive strength results (MPa).

Mix ID	7 days		28 days	
	Standard deviation	Coefficient of variation	Standard deviation	Coefficient of variation
Control	0.655744	6.071702	0.3	1.369863
Mix 1	0.351188	2.94292	0.360555	1.547447
Mix 2	0.404145	2.635729	0.378594	1.543182
Mix 3	0.251661	1.495017	0.264575	1.013698
Mix 4	0.378594	3.957427	0.493288	2.487168

**Figure 4:**
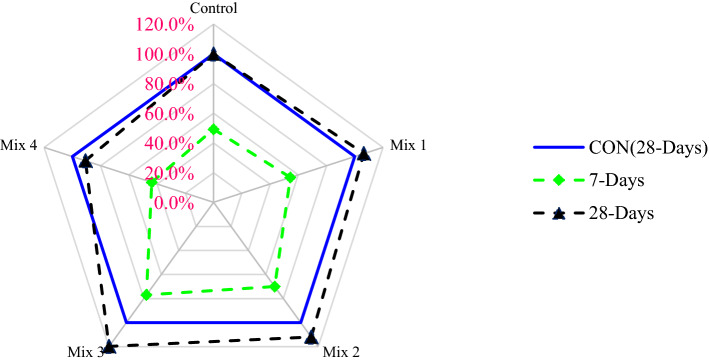
Relative analysis of compressive strength.

A relative analysis was carried out in which the curing of 28 days age of control mix compressive strength was considered the related mix, and from this, different blends with changing percentages are compared, as shown in Fig. 4. At 7 days of curing, compressive strength was about 23% less than that of the control (28 days) at 45% CS aggregate, 1.5% glass fibers, and 15% silica fume (optimum dosage). Compared to the control, 45% CS aggregate, 1.5% glass fibers, and 15% silica fume (optimum dosage) showed 20% higher compressive strength at 28 days of curing. Therefore, it is recommended to use 45% CS aggregate, 1.5% glass fibers, and 15% silica fume (optimum dosage) in concrete to make concrete with good compressive strength.

#### Splitting tensile strength

It is a technique of evaluating the concrete tensile strength using a cylinder. According to ASTM C496-71^29^, a split cylinder test was completed on cylindrical samples of 300 mm height and 150 mm diameter and at the ages of 7, and 28 days of curing.

**Figure 5:**
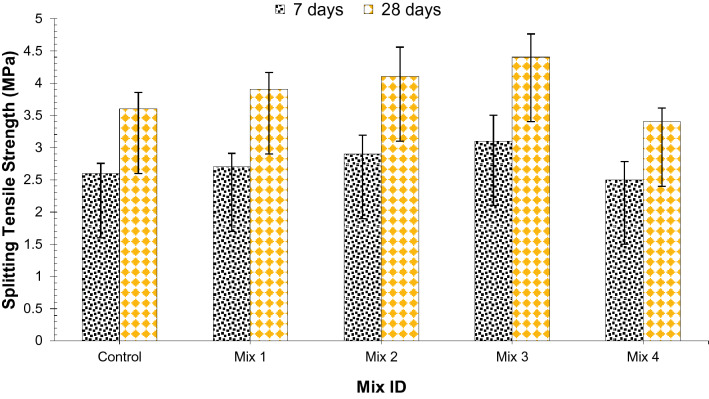
Splitting tensile strength of concrete.

Figure 5 presents the split tensile strength of different mixes while its standard deviation and coefficient of variation for 7 and 28 days is displayed in Table 8. Adding glass fibers to concrete impressively enhances the concrete flexural and split tensile attributes during the hardened stage, for example, rigidity, flexural strength, toughness, and flexibility^30^. Past examinations have demonstrated that the consideration of fibers fundamentally enhances the split tensile strength of lightweight aggregate concrete^31,32^. In the present examination, the addition of glass fibers and silica fume to coconut Shell concrete applies a gainful impact on split tensile and flexural strength. After the test, it was noted that concrete achieved its highest flexural and split tensile strength at 45% coconut shell aggregate, 1.5% glass fibers, and 15% silica fume. Although it has been revealed that surplus quantity of coconut shell aggregates reduces strength due to poor bond between cement paste resulting in porous concrete^13^. Increasing the percentages of the above materials reduced the concrete split and tensile flexural strength. Fibers are mixed in concrete to increase the flexibility of concrete by halting the onset of tension cracks or preventing the generation of cracks in such a manner that the tensile strength of (SFRC) steel fiber reinforced concrete displays better conduct than normal concrete. Fibers assists in dispensing the applied forces to the whole body of concrete. Fibers are known to enhance the tensile capacity of post-cracking behavior^21,33^. Fibers have shown more substantial effects on split tensile and flexural strength at 0.5 to 2.0 percent volume fractions added in the study^21,34^. Moreover, Silica fume particles are 100 times smaller than cement grains and so they can pack very well with cement grains. They also react with CH to form CSH which gives additional binding properties and results in strength increase. However, at higher dosage Mix 4 (60% CS aggregate, 2.0% glass fibers, and 20% silica fume), the workability of concrete decreases, because the size of silica fume is so small, when we add more silica fume it will increase the surface area and demand more water and if we add more water it will reduce the strength.

**Table 8:** Standard deviation and coeffiecient of variation of split tensile strength results (MPa).

Mix ID	7 days		28 days	
	Standard deviation	Coefficient of variation	Standard deviation	Coefficient of variation
Control	0.152753	5.951397	0.251661	6.926454
Mix 1	0.208167	7.806247	0.264575	6.783978
Mix 2	0.288675	9.954315	0.458258	11.08688
Mix 3	0.4	12.90323	0.360555	8.132822
Mix 4	0.282843	11.31371	0.212132	6.239177

**Figure 6:**
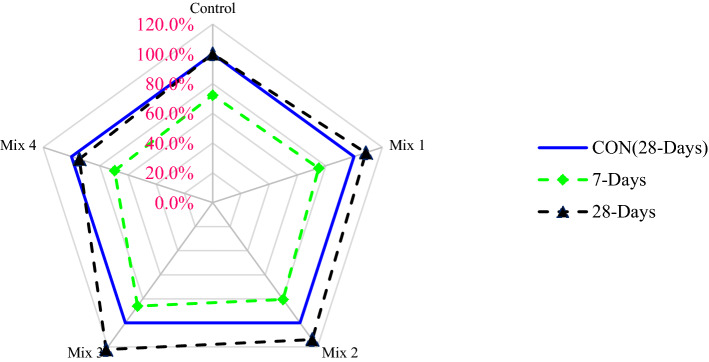
Relative analysis of splitting tensile strength.

A relative analysis was carried out in which the curing age of the 28-days control sample split tensile strength was considered the related mix, and from this, different blends with changing percentages are compared, as shown in Fig. 6. At 7 days of curing, split tensile strength was about 14% less than that of the control (28 days) at 45% CS aggregate, 1.5% glass fibers, and 15% silica fume (optimum dosage). Compared to the control, 45% CS aggregate, 1.5% glass fibers, and 15% silica fume (optimum dosage) showed 22% higher spilt tensile strength at the curing age of 28 days. Therefore, to make concrete with good split tensile strength it is recommended to used 45% CS aggregate, 1.5% glass fibers, and 15% silica fume (optimum dosage) in concrete.

Comparison of predicted values with experimental tensile strength values utilizing ACI-318.11 codes is displayed in Fig. 6. Equation (1) can be used to predict the values of split tensile strength from compressive strength.

1 $$ f{\text{sp}} = {\text{~}}0.53 \times {\text{~}}\sqrt {f{\text{c}}} $$

**Figure 7:**
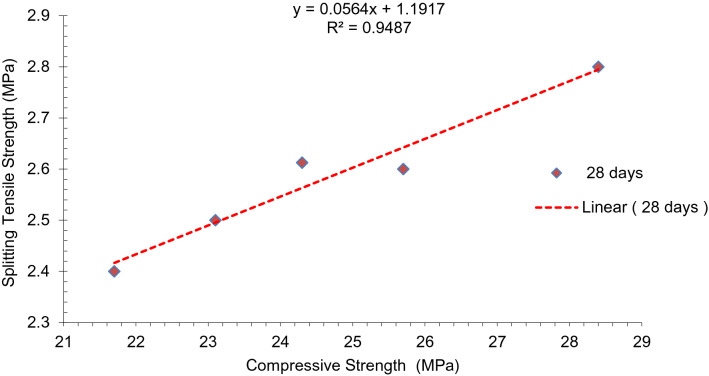
Co-relation between splitting tensile strength and compressive strength.

It is observed that entire empirical values locate well in anticipated values utilizing ACI-318.11 codes. Regression models between experimental values of split tensile strength and compressive strength are displayed in Fig. 7. A strong correlation occurs (R^2^ > 0.94) amid both strengths.

### Durability tests of concrete

#### Density

Density is an indirect method to determine the durability of concrete i.e. higher density gives more dense concrete leading to fewer voids resulting in more durable concrete. Concrete density with varying percentages of different dosages was determined as per ASTM C138^35^.

**Figure 8:**
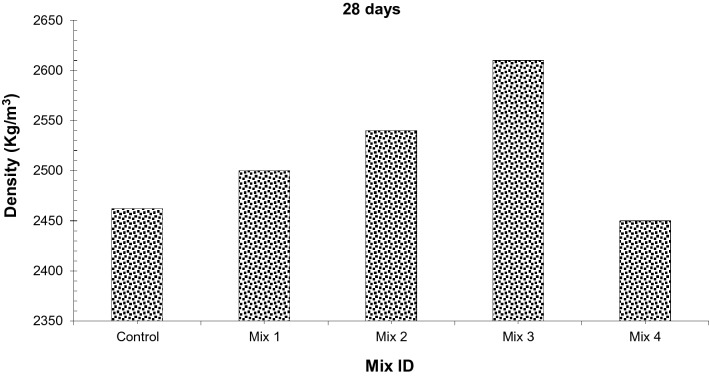
Density of concrete.

Figure 8 shows the density of concrete with different dosages. It can be observed that concrete density increases up to Mix 3 (45% CS aggregate, 1.5% glass fibers, and 15% silica fume) and then decreases gradually having a maximum density at Mix 3 (45% CS aggregate, 1.5% glass fibers, and 15% silica fume) while minimum density is obtained at Mix 4 (60% CS aggregate, 2.0% glass fibers, and 20% silica fume). Although it has been reported that excess amount of coconut shell aggregates reduces strength due to poor bond between cement paste resulting in porous concrete. Fibers controls and constrain the development of cracks in concrete caused both in the plastic and hardened stage of concrete hence confirming a more durable concrete^30,31,33^. Furthermore, silica fume enhances density due to pozzolanic reaction i.e. giving secondary C–S–H gel which rises the paste viscosity leading to more packed concrete. However, mix 4 (60% CS aggregate, 2.0% glass fibers, and 20% silica fume), shows less density than the control specimen. The density of coconut shell aggregate is less than coarse aggregate, and if we add more quantity of coconut shell aggregate it will lead to concrete with less strength than the control specimen.

**Figure 9:**
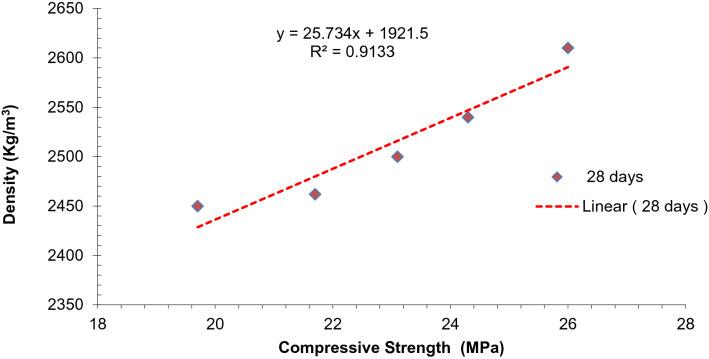
Co-relation between density and compressive strength.

As density directly affects strength. Higher density gives more dense concrete leading to less void which ultimately increases strength. Therefore, a strong correlation is existing between density and compressive strength. The correlation between compressive strength and density is displayed in Fig. 9. It can be observed that the regression line between compressive strength and density is appeared to be linear. Regression modal shows a strong co-relation between compressive strength and flexure strength having an R^2^ value greater than 90%.

#### Ultrasonic pulse velocity (UPV) test

**Figure 10:**
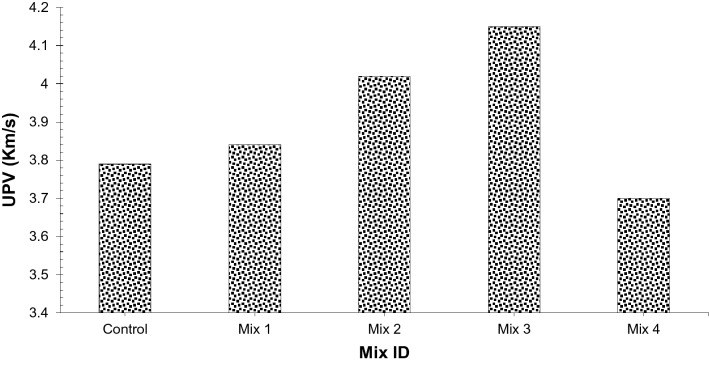
UPV test results.

It is a non-destructive test performed to evaluate concrete integrity and homogeneity as per ASTM C 597-02^32^. Subjective evaluation of concrete strength and degree of concrete in various areas of members of structure might be accomplished by utilizing this technique. Any depth of surface cracks, concrete cover examination, lack of coherence in the cross-section (for example cracks can additionally be determined). UPV test of all mixes was estimated, and, the relationship between the UPV and compressive strength of CS concrete was calculated at 28 days as shown in Fig. 10. Concrete is sound an 'acceptable' state when its ultrasonic pulse velocity values are in the middle of 3.70 km/s and 4.61 km/s^36^. Whole concrete samples were exposed to the Ultrasonic Pulse Velocity test earlier than the destructive tests. Overall, the values of the ultrasonic pulse velocity Test of entire blends improved with rising compressive strength. The values of the ultrasonic pulse velocity test entire blends are between 3.76 and 4.21 km/s. So, it can be observed from the test, that the coconut shell concrete with glass fibers have more ultrasonic pulse velocity values.

**Figure 11:**
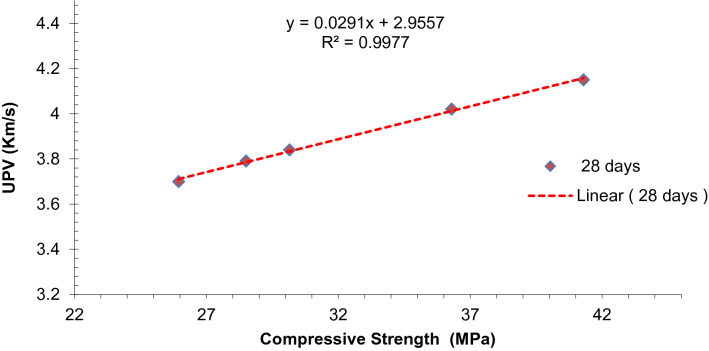
Co-relation between UPV and compressive strength.

Ultrasonic pulse velocity calculations corresponding to their related compressive strength are shown in Fig. 11. Equation (2) can be used to predict values of ultrasonic pulse velocity from compressive strength.

2 $$ f{\text{sp}} = {\text{~}}fck = 0.{\text{13}}v^{{{\text{4}}.0{\text{481}}}} $$

*fck* represents compressive strength in (Mpa), and *v* is the UPV (km/s).

Regression models between Ultrasonic Pulse Velocity and experimental values of compressive strength show a strong correlation having R^2^ greater than 90 percent.

#### Water absorption

Water absorption is an indirect measurement of concrete durability. Mostly harmful chemicals are present in water. These chemicals react with concrete ingredients, which changes the properties of concrete. Extra water present in the pores of concrete results in freezing and thawing cycles effect because of the change in temperature, which results in a concrete crack. Therefore, a water absorption test was conducted on all samples at 7 and 28 days.

**Figure 12:**
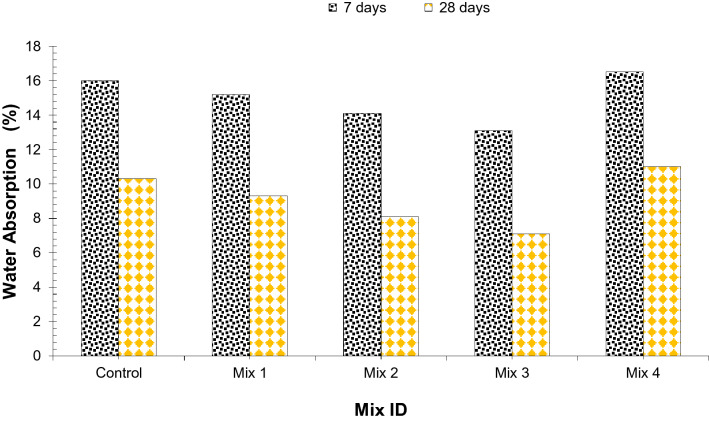
Water absorption of concrete at 7 and 28 days of curing.

Water absorption test results are displayed in Fig. 12. A general trend indicates that water absorption decreases up to Mix 3 (45% CS aggregate, 1.5% glass fibers, and 15% silica fume) and then increases gradually at Mix 4 (60% CS aggregate, 2.0% glass fibers, and 20% silica fume). Although it has been reported that coconut shell aggregate reduces strength due to the poor bond between CS aggregate and cement paste resulting in porous concrete which increases water absorption^13^. Fibers behave as crack arresters and don’t prevent cracks which prevent cracks from propagation in the concrete leading to more strong concrete resulting in less water absorption^33^. Furthermore, silica fume enhances density due to pozzolanic reaction i.e. giving secondary CSH gel which enhances the paste viscosity that results in more dense concrete which also contributes to decreasing water absorption. However, mix 4 (60% CS aggregate, 2.0% glass fibers, and 20% silica fume), shows more water absorption than the reference sample. the porosity of coconut shell aggregate is more than coarse aggregate, and if we add more quantity of coconut shell aggregate it will absorb more water than it should, and surplus water in the concrete is not good and it leads to multiple issues like honeycombing in concrete, porous concrete, and concrete with less strength and durability, hence it is recommended to utilized optimum quantity (45%) of coconut shell aggregate in concrete to make concrete suitable for structural application and eco-friendly.

#### Acid resistance test

**Figure 13:**
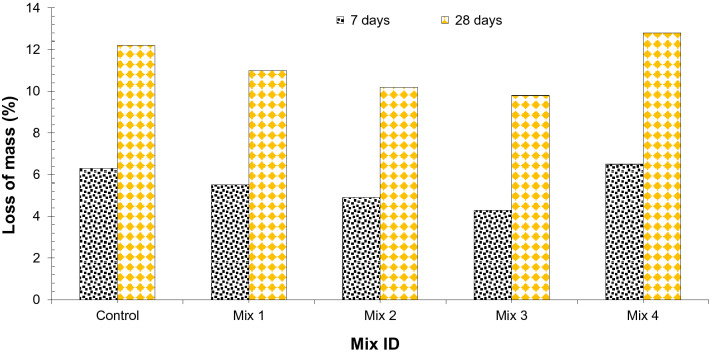
Acid resistance of concrete at 7 and 28 days in acid.

Strong acids can be found in different varieties, for example, nitric acid, hydrochloric acid, acetic acid, and sulfuric acid (H_2_SO_4_), etc. In this study, Sulfuric acid was taken as an acid strike, on a concrete sample with different dosages. The test outcome after exposure to acid is shown in terms of mass loss because of the H_2_SO_4_ attack on the specimens after 7 and 28 days for each blend as shown in Fig. 13. It can be noted that weight loss due to sulfuric acid considerably decreases up to Mix 3 (45% CS aggregate, 1.5% glass fibers, and 15% silica fume) and then increase gradually having maximum loss at Mix 3 Mix 4 (60% CS aggregate, 2.0% glass fibers, and 20% silica fume). Erosion of concrete is the dissolution of calcium aluminate and calcium hydroxide due to sulfuric acid^35,37,38^. Erosion speed will largely depend on sulfuric acid penetration rate into the concrete body and to reach calcium aluminate and calcium hydroxide. Therefore, improvement in the concrete porosity leads to increased density of concrete because of the addition of glass fibers. The rise in density would lead to less penetration rate of sulfuric acid in concrete. Although it has been reported that coconut shell aggregate reduces strength because of the poor bond between CS aggregate and cement paste resulting in porous concrete which decreases density^13^. Fibers behave as crack stoppers and not as cracks prevention which decreases void in hardening concrete leading to more dense concrete resulting in more dense concrete^21,33^. However, mix 4 (60% CS aggregate, 2.0% glass fibers, and 20% silica fume), shows more weight loss than the control/ blank mix. It is because at higher dosage (60% CS aggregate, 2.0% glass fibers, and 20% silica fume), the workability of concrete decreases which increases compaction efforts resulting porous concrete leading to less density which ultimately increases loss of the weight.

## SEM observations

**Figure 14:**
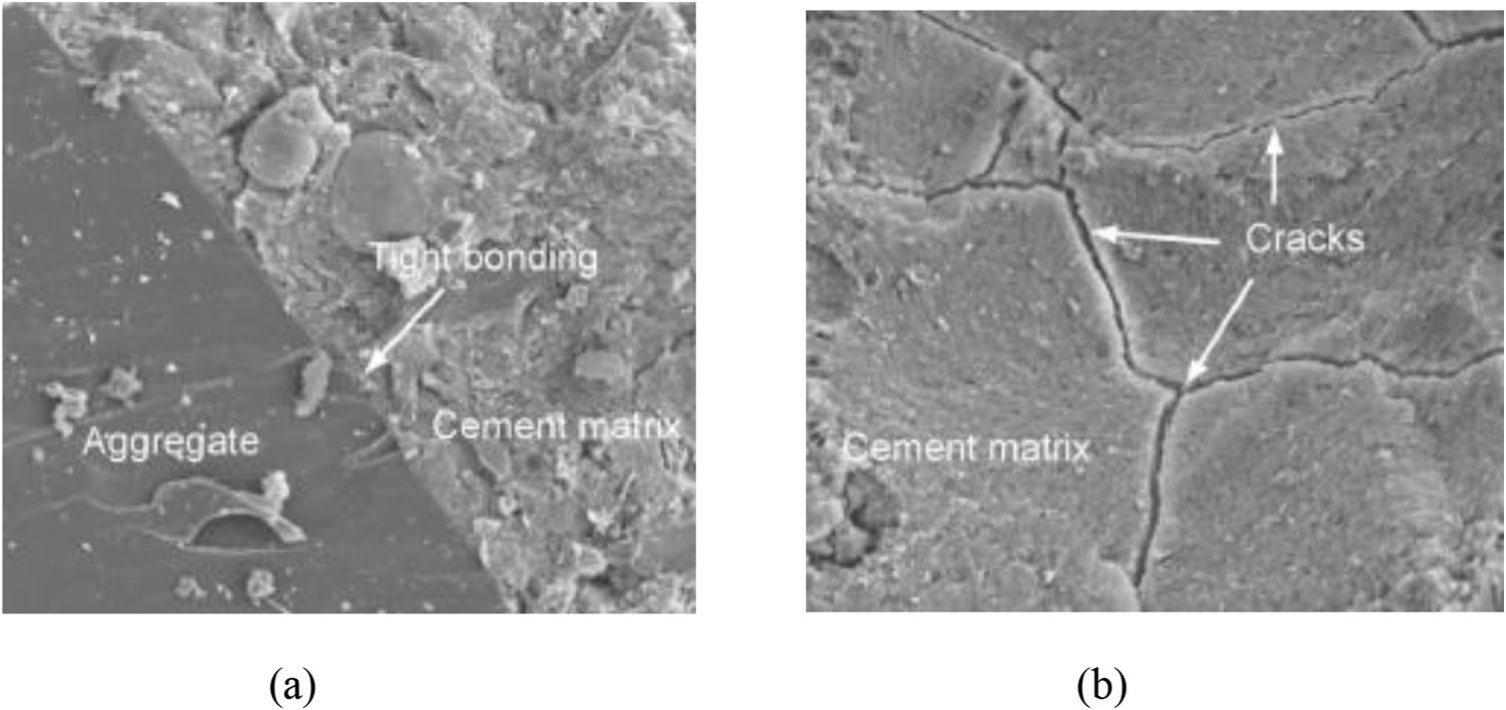
(**a**, **b**) SEM Images of control concrete.

The scanning electron microscopic test was conducted after 28 days of curing as per ASTM C1723-2010 on the control concrete sample and concrete sample with glass fibers, in it. Figure 14a exhibits the SEM images of the control concrete sample, which show a good quality bond is present between binder matrix and aggregate, and the binder matrix is very compact. Yet, in Fig. 14b clear cracks in the binder matrix can be noted.

**Figure 15:**
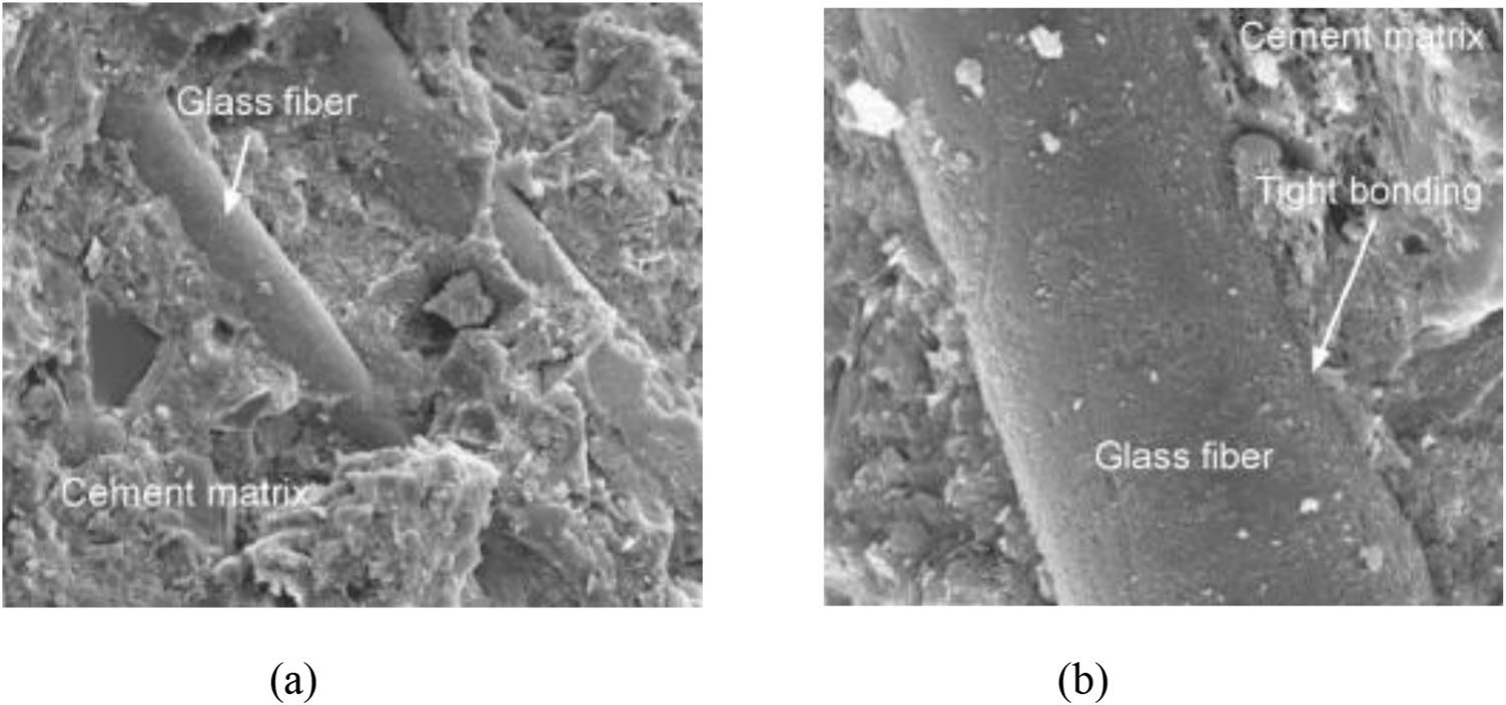
(**a**, **b**) SEM Images of concrete with glass fibers.

Figure 15a shows the SEM images of the concrete sample with glass fibers, silica fume, and recycled aggregate in it. As a multi-phase, multi-interface, and multi-element composite material, glass fiber-reinforced concrete behavior mostly relies on the properties between the binder matrix and the glass fiber. It could be noted from Fig. 15a, b that binder matrix and glass fiber are very strongly bonded, which can be clarified by the fact that glass fiber has improved hydrophilicity and also mineral fiber material. The quality of bonding between binder-matrix and glass fibers provides enhanced strength and also reduced the water absorption of the concrete. This is also because glass fibers played a filling role and decreased the concrete porosity leading to denser and less porous concrete.

## Sustainability performance

Substitution of coarse aggregate with coconut shell aggregate in concrete not just preserves granite stone, which is the quickest draining common resource yet besides answer for waste coconut shell waste removal. In concrete, when silica fume is utilized, the depletion of limestone, a major ingredient needed for the making of cement is lessened and thus the angle of sustainability in the production of concrete is achieved. It shall altogether help in preserving the limestone, furthermore, effectively decrease the release of nitrogen dioxide, carbon dioxide, and more harmful materials into the climate. It can be noticed that the utilization of fiber-reinforced concrete has mechanical performance, made with the joint adding of glass fibers, coconut shell, and silica fume, and it could be utilized for building structure use. Therefore, in concrete production sustainable development could be accomplished by the utilization of glass fibers, waste coconut shells, and silica fume.

## Conclusions

The effects of utilizing coconut shell aggregate, glass fibers, and silica fume at different percentages on the mechanical attributes and durability aspects of waste CS concrete are considered. From the trial examination, the following interpretations are obtained.

- Coconut shell concrete compressive strength increases about 20% strength at 45% CS aggregate, 1.5% addition of glass fiber, and 15% silica fume at 28 days.
- At 45% substitution of coconut shell aggregate with crushed aggregate and 1.5% addition of glass fiber and 15% silica fume, the concrete split tensile strength increases 22% at 28 days.
- Substitution of conventional crush by coconut shell forms lighter concrete. Unit weight of concrete decreased roughly 21.97% for 60% of CS aggregate substitution, which can decrease structure dead load. Hence, installation, erection, and footing costs can be reduced.
- The density of coconut shell concrete enhanced up to the optimum level and also shows from the co-relation between compressive strength and density that CS concrete is a doable option.
- UPV test of coconut Shell concrete showed a concrete with good integrity and homogeneity, which was also proved from the co-relation between compressive strength and UPV test.
- When glass fibers were added to coconut shell concrete, it displayed good behavior under the acid resistance test, because glass fibers reduced the concrete porosity and improved concrete density due to which less acid penetrated the concrete, that proves that this concrete can be used in structural applications.
- Concrete mechanical strength was significantly improved due to addition of glass fibers and silica fume. Glass fibers provided confinement effect to the concrete and silica fume made extra C–S–H which leads to more strength.
- From the scanning electron microscope (SEM) test, it was revealed when the glass fibers are added to concrete, it improves the bonding between the binder matrix and the glass fibers leading to denser and less porous concrete. Glass fibers also prevented the cracks from propagation in concrete hence increasing concrete mechanical properties.

Using only coconut shell as coarse aggregates in replacement of crushed aggregates may result in mediocre strength concrete with mediocre durability properties, now using glass fibers and silica fume which are both industrial waste materials or by-products will significantly enhance concrete strength, and will not cost as much, and helps the environment by effectively utilizing waste products. Coconut shell glass-fiber-reinforced concrete with silica fume is a reasonable option to be used as a feasible construction material that is sustainable in the making of concrete for structural applications, which is environmentally friendly, low cost, and has good strength.

## Data Availability

The data required to support the present findings are present in the manuscript.
